# Bias in context: What to do when complete bias removal is not an option

**DOI:** 10.1073/pnas.2304710120

**Published:** 2023-05-30

**Authors:** Sune Holm, Eike Petersen, Melanie Ganz, Aasa Feragen

**Affiliations:** ^a^Department of Food and Resource Economics, University of Copenhagen, Frederiksberg 1958, Denmark; ^b^Pioneer Centre for AI, Copenhagen 1350, Denmark; ^c^Denmark Technical University (DTU) Compute, Kongens, Lyngby 2800, Denmark; ^d^Department of Computer Science, University of Copenhagen, Copenhagen 2100, Denmark; ^e^Neurobiology Research Unit, Copenhagen University Hospital, Copenhagen 2100, Denmark

It is widely recognized that machine learning algorithms may be biased in the sense that they perform worse on some demographic groups than others. This motivates algorithmic development to remove algorithmic bias, which in turn might lead to a hope—even an expectation—that algorithmic bias can be mitigated or removed (1). In this short comment, we make three points to qualify Wang et al.’s suggestion: 1) It may not be possible for algorithms to perform equally well across groups on all measures, 2) which inequalities count as morally unacceptable bias is an ethical question, and 3) the answer to the ethical question will vary across decision contexts.

## 1. Complete Bias Mitigation May Not Be Possible with the Data at Hand

Wang et al. describe how neuroimaging AI models are biased by having “different area under the curve (AUCs) for different genders, age, and racial groups,” and show how such bias can be reduced via robust training. However, different notions of algorithmic fairness can be at odds with each other (2), and mitigating bias with respect to one notion of fairness can lead to increasing bias with respect to another. In particular, bias caused by differences in data representation or quality (3, 4) is unlikely to disappear due to better training.

## 2. What Bias to Mitigate Is an Ethical Question

Wang et al. define unacceptable bias as inequality across groups with respect to model performance as measured by AUC. If we want to generalize this choice to other applications, we need to consider whether performance inequalities in general are morally acceptable. However, whether a form of performance inequality is morally problematic is an ethical question (5). If we want to claim mitigation of bias—in a morally problematic sense—we thus need to first provide an argument for why a particular measure—such as AUC—ought to be equalized. Moreover, if achieving equality in AUC is incompatible with achieving equality in another measure, such as accuracy, then designers should provide some justification for this priority.

## 3. What Counts as Morally Bad Bias Depends on Context

One might think that if AUC inequality is a morally unacceptable bias with respect to a neuroimaging algorithm, then it will be unacceptable for other algorithms as well. Still, this seems implausible. Which inequalities are of moral concern will necessarily be context dependent. For example, one might argue that when it comes to prescreening cardiovascular diseases, one important measure to equalize is true-positive rate in order to never miss a potential future stroke, whereas when deciding on an invasive follow-up procedure, false positives may also play an important role and equalizing another measure such as AUC might be more appropriate.

## 4. Conclusion

To conclude: While diagnosing and mitigating bias is of great importance in medicine, we warn against establishing fixed recommended metrics for measuring fairness. Instead, when assessing bias in a medical imaging context, developers need to have the clinical context in mind and evaluate algorithms in their use-context in order to justify their characterization of morally unacceptable bias.
